# Intra- and interrater reliability of the Modified Ashworth Scale and its association with the Tardieu Scale in children with cerebral palsy

**DOI:** 10.7717/peerj.21349

**Published:** 2026-07-01

**Authors:** Daša Mikša Pušnik, Sergej Pirkmajer, Tina Tomc Z˘argi

**Affiliations:** 1 Community Health Centre Ljubljana, Ljubljana, Slovenia; 2 Institute of Pathophysiology, Faculty of Medicine, University of Ljubljana, Ljubljana, Slovenia; 3 Department of Physiotherapy, Faculty of Health Sciences, University of Ljubljana, Ljubljana, Slovenia

**Keywords:** Children, Cerebral palsy, Spasticity assessment, Psychometric properties, Modified Ashworth scale, Tardieu scale

## Abstract

**Background:**

The Modified Ashworth Scale is a widely used tool for assessing spasticity. The aim of this study was to evaluate the intra- and interrater reliability of the Modified Ashworth Scale and its association with the Tardieu Scale in children with cerebral palsy.

**Methods:**

In this cross-sectional study, 23 children with cerebral palsy were included. The intra- and interrater reliability of the Modified Ashworth Scale was assessed for the elbow, knee, and plantar flexors of the foot. The results of the Tardieu Scale were included to evaluate the association between the two scales. The intraclass correlation coefficient (ICC) was used to assess reliability, and Spearman’s correlation coefficient was used to assess the association between the Modified Ashworth Scale and the Tardieu Scale. All muscles assessed were included in the analysis, regardless of the anatomical classification of physical impairment.

**Results:**

Intrarater reliability (ICC) in our sample was excellent for all muscles assessed (ICC = 0.91–0.99). Interrater reliability was generally good, with confidence intervals indicating moderate to good reliability in certain muscle groups (ICC = 0.80–0.89). A moderate positive correlation was observed between the Tardieu Scale and the Modified Ashworth Scale for all muscles assessed.

**Conclusions:**

The results demonstrated excellent intrarater and generally good interrater reliability in a heterogeneous primary healthcare sample, with a moderate positive correlation between the scales studied. However, caution is warranted when interpreting findings at the individual level.

## Introduction

Cerebral palsy (CP), also known as static encephalopathy, results from permanent, non-progressive impairment of the developing brain (Rosenbaum et al., 2007; Swaiman & Packer, 2011). Brain lesions in children with CP are heterogeneous; however, all forms are characterized by pathological patterns of movement and posture (Rosenbaum et al., 2014). The spastic (pyramidal) form of CP, which occurs in most cases, may present with negative or positive neurological features (Aravamuthan & Waugh, 2016; Graham et al., 2016), including altered muscle tone. In healthy individuals, regulation of muscle tone enables coordinated movement and postural control. Muscle stretch elicits a reflex increase in muscle tone, preventing excessive or unexpected excursion (Nielsen et al., 2005). The stretch reflex operates as feedback involving intrafusal muscle fibers within the muscle spindle, responding both to static changes in muscle length (the static component of the reflex) and to the rate of change in muscle length (the phasic component of the reflex) (Clarac, Cattaert & Le Ray, 2000). In the early stages of CP, alterations are predominantly functional, including hyperexcitability of the stretch reflex, which contributes to positive motor signs such as spasticity (Li & Francisco, 2015). Spasticity is defined as a motor disorder characterized by a velocity-dependent increase in resistance during passive stretching or as involuntary muscle activity (Sanger, 2003).

The Modified Ashworth Scale (MAS) is a clinical tool used to assess spasticity by grading the resistance felt during rapid passive movement of a limb (Mutlu, Livanelioglu & Gunel, 2008; Pandyan et al., 2001). The MAS employs a six-point ordinal scale (0, 1, 1+, 2, 3, 4), where 0 indicates no increase in tone and 4 indicates rigidity. During assessment, the examiner moves the limb from the maximally shortened to the maximally stretched position over approximately one second (Bohannon & Smith, 1987).

The MAS is quick to administer, requires no additional equipment and is widely used in clinical practice. Examiner experience has not consistently been shown to significantly affect reliability (Mutlu, Livanelioglu & Gunel, 2008; Pandyan et al., 1999). However, the MAS does not differentiate between neural and mechanical contributors to increased resistance. In the presence of muscle shortening or contracture, spasticity may therefore be overestimated (Gracies, 2005). The assessment is performed during passive movement, as the antagonistic muscle may limit the force of the tested agonistic muscle during active movement, and to avoid excessive stretch reflex facilitation (Pandyan et al., 2001; Bohannon & Smith, 1987; Lance, 1980).

To minimize stretch-induced inhibition of spasticity, no more than three passive movements are recommended per joint (Clopton et al., 2005). Even brief stretching may influence tone assessment (Bohannon & Smith, 1987). Despite its widespread use, concerns remain regarding the availability of reliable and accessible tools for spasticity assessment in children with CP at the primary healthcare level (Pandyan et al., 1999; Gracies et al., 2010). As spasticity assessment is central to evaluating therapeutic interventions, it is important to identify tools that are both feasible and reproducible. The Tardieu Scale (TS), first described in 1954 and subsequently modified, has been proposed as an alternative approach to spasticity assessment. The TS evaluates both the quality of muscle reaction and the difference between joint angles measured during slow and fast passive stretch, thereby capturing the velocity-dependent component of the stretch reflex (Gracies et al., 2010). The initial stretch is applied as slowly as possible, followed by a fast stretch, typically approximating the speed of segmental fall under gravity (Gracies et al., 2010). The TS is considered to better differentiate neural spasticity from soft tissue contracture compared to the Ashworth scale or MAS.

Whenever different assessment scales or different versions of the same scale (*e.g.*, Ashworth Scale, MAS, Modified MAS) are used in clinical practice or research, it is important to examine their association, as these scales assess related but distinct constructs. Without such verification, scores cannot be meaningfully compared, which hinders clinical decision-making and reduces comparability between studies. Although intra- (Mutlu, Livanelioglu & Gunel, 2008; Clopton et al., 2005; Klingels et al., 2010; Fosang et al., 2003; Salem et al., 2010; Numanoğlu & Günel, 2012) and interrater reliability of the Ashworth Scale or MAS (Mutlu, Livanelioglu & Gunel, 2008; Clopton et al., 2005; Klingels et al., 2010; Fosang et al., 2003; Salem et al., 2010; Numanoğlu & Günel, 2012) for various muscle groups has been widely investigated, the association between the MAS and the TS requires further examination. Previous studies have primarily compared the modified TS with MAS or modified MAS, or included patients with other neurological conditions such as stroke rather than children with CP (Numanoğlu & Günel, 2012; Yoo et al., 2022). The classic MAS uses a six-point ordinal scale (0, 1, 1+, 2, 3, 4), whereas the Modified MAS omits the 1+ category, resulting in a five-point scale (0–4). The Modified TS retains the original TS grading system but introduces procedural standardization, including defined limb positioning and alignment.

Although existing evidence suggests a possible association between TS and MAS in children with spastic CP in primary outpatient settings, this relationship has not been directly demonstrated. Furthermore, there remains a need for accessible and feasible assessment tools suitable for use by therapists in primary healthcare.

The aim of this study was therefore to examine the applicability of MAS in this specific clinical context. Specifically, we assessed the intra- and interrater reliability of MAS and the association between MAS and TS in children with CP receiving neurodevelopmental physiotherapy in a primary healthcare setting.

## Materials and Methods

This cross-sectional reliability and association study was conducted by three physiotherapists (examiners A, B, and C). Examiners B and C had more than ten years of experience treating children and adults with developmental disabilities, while examiner A (the lead investigator) had less than three years of experience. The study included 23 children with CP of both sexes (aged 6–14 years) who had previously been classified according to the Gross Motor Function Classification System (GMFCS) (levels I–IV) by an attending pediatrician at the Community Health Centre. Inclusion criteria were the presence of spasticity; no botulinum toxin injections or corrective musculoskeletal surgical procedures involving the examined muscles within the previous six months; no history of dorsal rhizotomy; and the ability to follow instructions.

Participants were recruited from the outpatient population receiving therapeutic treatment at the institution, irrespective of etiology or timing of impairment. All children attended the same outpatient clinic and received treatment at comparable frequencies from therapists trained in neurodevelopmental physiotherapy. Given the variability in functional severity among children with spastic CP attending primary outpatient units in our country, the sample was considered reflective of the target clinical population in which the MAS is routinely applied. Table 1 summarizes participant characteristics.

**Table 1:** The clinical characteristics of included children.

Sex, girl/boy (n)	9 (39%)/14 (61%)
Age range (years), mean (SD)	6–14, 10.6 (2.7)
Pathophysiological classification of CP	
Spastic	23 (100%)
Anatomical classification of movement impairment	
Side of mobility impairment, left/right	14 (61%)/9 (39%)
Hemiparesis	18 (78,3%)
Diplegia	3 (13%)
Tetraparesis	2 (8,7%)
Level of GMFCS	
I	17 (74%)
II	3 (13%)
III	1 (4,3%)
IV	2 (8,7%)

**Notes.**

CP cerebral palsy GMFCS Gross motor function classification system

Ethical approval was obtained from the Medical Ethics Committee of the Community Health Centre (approval code: 852-1/2019-3, approved on 7 May 2019). Of 26 invited participants, 24 provided informed consent. One child was later excluded from analysis due to a dyskinetic form of CP. Participants and their legal representatives received verbal and written information prior to enrolment and signed informed consent forms. Personal identifiers were replaced with coded numbers before analysis, and data were stored securely with access restricted to authorized personnel.

### Measurements

Before data collection, the examiners familiarized themselves with the assessment scales and jointly evaluated one participant to standardize testing procedures and clarify any uncertainties. Approximately one month later, this participant was included in the study. Each participant was assessed twice, seven to fourteen days apart, to minimize potential recall effect from the initial assessment. During the first measurement session, all three examiners independently performed MAS assessments at five-minute intervals in random order. Assessments were conducted in separate rooms, examiners were blinded to each other’s scores, and results were recorded immediately and concealed to prevent access to prior or concurrent assessments. Five minutes after completion of all MAS assessments, the lead examiner performed the TS assessment. During the second measurement session, only the lead examiner repeated the MAS assessment. Intrarater reliability was calculated using the lead examiner’s results from the first and second sessions, whereas interrater reliability was determined using only the first measurement.

The participant used during examiner familiarization was not excluded from the analysis, as the training session occurred approximately one month prior to formal data collection. This interval was considered sufficient to minimize potential recall or learning effects. Additionally, the familiarization session focused on standardization of testing technique rather than scoring calibration for that specific individual.

### Testing procedure

To assess spasticity of the elbow, knee and plantar flexors, subjects were placed in the supine position on a hard, padded surface (Airex^®^ mat). If participants were unable to stabilize the head, it was maintained in the neutral position and the arms were placed beside the body in supination. The neutral head position was maintained throughout the procedure to inhibit tonic neck reflexes. To assess spasticity of the knee flexors, the hip on the examined side was flexed to 90°, and the contralateral knee was flexed sufficiently for the foot to rest on the floor. When assessing soleus muscle spasticity, the knee on the examined side was supported by a padded cylinder (15 × 60 cm). Participants were barefoot, without splints, and wore a T-shirt and shorts during the measurements. Participants were instructed to remain relaxed. Relaxation was facilitated through standardized positioning and handling procedures. Limbs were supported proximally, rapid preparatory movements were avoided, and testing commenced only when the limb appeared visually and palpably relaxed. Sensory stimulation was minimized, and head and trunk positioning were carefully controlled to avoid reflex facilitation.

The MAS test was performed three times over the available range of motion, one second apart, from the maximally shortened to the maximally stretched muscle position. The passive stretch was applied at a velocity corresponding to one second per movement (Bohannon & Smith, 1987). Before data collection, all examiners jointly tested the assessment procedure to standardize protocol and passive movement velocity by assessing one participant together approximately one month in advance. As a sensitivity analysis, all primary statistics were recalculated with this participant excluded; results were not materially altered.

The examiner stabilized the proximal segment (upper arm, outer thigh, or ankle over the Achilles tendon) and applied movement distally. Stabilization points and hand placements were predefined and standardized across examiners. The MAS outcome was defined as the average of three measurements (Bohannon & Smith, 1987; Clopton et al., 2005). MAS grades (0, 1, 1+, 2, 3, 4) were applied according to the original operational definitions described by Bohannon & Smith (1987) and clarified by Pandyan et al. (1999), without modification. For statistical analysis, MAS grades were converted into numerical values (0 = 0, 1 = 1, 1+ = 1.5, 2 = 2, 3 = 3, 4 = 4), consistent with previous studies. Numerical conversion was applied before averaging. For each muscle and examiner, three repeated measurements were obtained, converted to numerical values, and the final MAS score was calculated as the arithmetic mean of these three repetitions. This averaged score was used in all subsequent analyses, including ICC, Bland–Altman, and correlation analyses. The exact grade definitions used in this study are presented in the Appendix (Table S1).

The TS assessment included two variables: the angle of muscle reaction and the quality rating of spasticity. Spasticity was operationalized as the difference between passively measured joint angles at high and low velocity (R2–R1) (Gracies et al., 2010). Passive joint angles were measured using a manual plastic goniometer with a 360°  scale (Baseline™ goniometer). During elbow extension measurement, participants remained supine. Knee extension was measured with the hip flexed to 90°  and the contralateral knee flexed to allow foot contact with the floor. For assessment of gastrocnemius spasticity, ankle dorsiflexion was measured with the knee extended. For assessment of soleus spasticity, the knee of the examined side was supported with a padded cylinder. The number of examiners, stabilization technique, and goniometer positioning remained standardized throughout measurements. If no palpable catch or clonus was observed during high-velocity stretch (TS quality score 0 or 1), the angle of catch could not be determined. In these cases, a value of 0 degrees was assigned to represent the absence of a measurable velocity-dependent response (Gracies et al., 2010). All measurements were performed during morning hours. Participants received verbal and written instructions to be well rested before assessment and to avoid unusually strenuous physical activity within 24 h before testing.

### Data analysis

Reliability and association analyses were conducted using IBM SPSS Statistics 26.0 (IBM Corp., Armonk, NY).

Intrarater reliability was assessed using a two-way mixed-effects model with absolute agreement and average measures (ICC(3,k)), as repeated measurements were performed by the same examiner, who was considered a fixed effect. Interrater reliability was assessed using a two-way random-effects model with absolute agreement and average measures (ICC (2,k)), as examiners were considered a random sample of clinicians and results were intended to be generalizable to similar clinical settings, in accordance with the guidelines of Koo & Li (2016). Average measures (k) were used because MAS scores were calculated as the mean of three repetitions, reflecting routine clinical practice (Clopton et al., 2005).

Although the MAS is an ordinal measure, ICC was applied in line with the predominant methodological approach in MAS reliability studies to allow comparison with previous literature. To examine the association between the MAS and the TS, Spearman’s rank correlation coefficient was calculated, as it is appropriate for ordinal data and does not assume normal distribution (Mukaka, 2012).

In addition to ICC, Bland–Altman analysis was performed to further evaluate interrater agreement and detect potential systematic bias between examiners. For each muscle group and examiner pair, mean differences (bias), 95% confidence intervals of the mean differences, and 95% limits of agreement (LoA) were calculated. Limits of agreement were defined as the mean difference ±1.96 standard deviations of the differences. Given the ordinal nature of MAS, Bland–Altman results were interpreted cautiously as supplementary exploratory analyses.

All examined muscles were included in the reliability analyses, irrespective of anatomical classification of motor impairment, as the aim was to evaluate agreement across a heterogeneous primary healthcare population.

## Results

Intrarater reliability was excellent across all examined muscle groups, with the highest agreement for elbow flexors and plantar flexors, and slightly lower but still excellent agreement for knee flexors.

**Figure 1:**
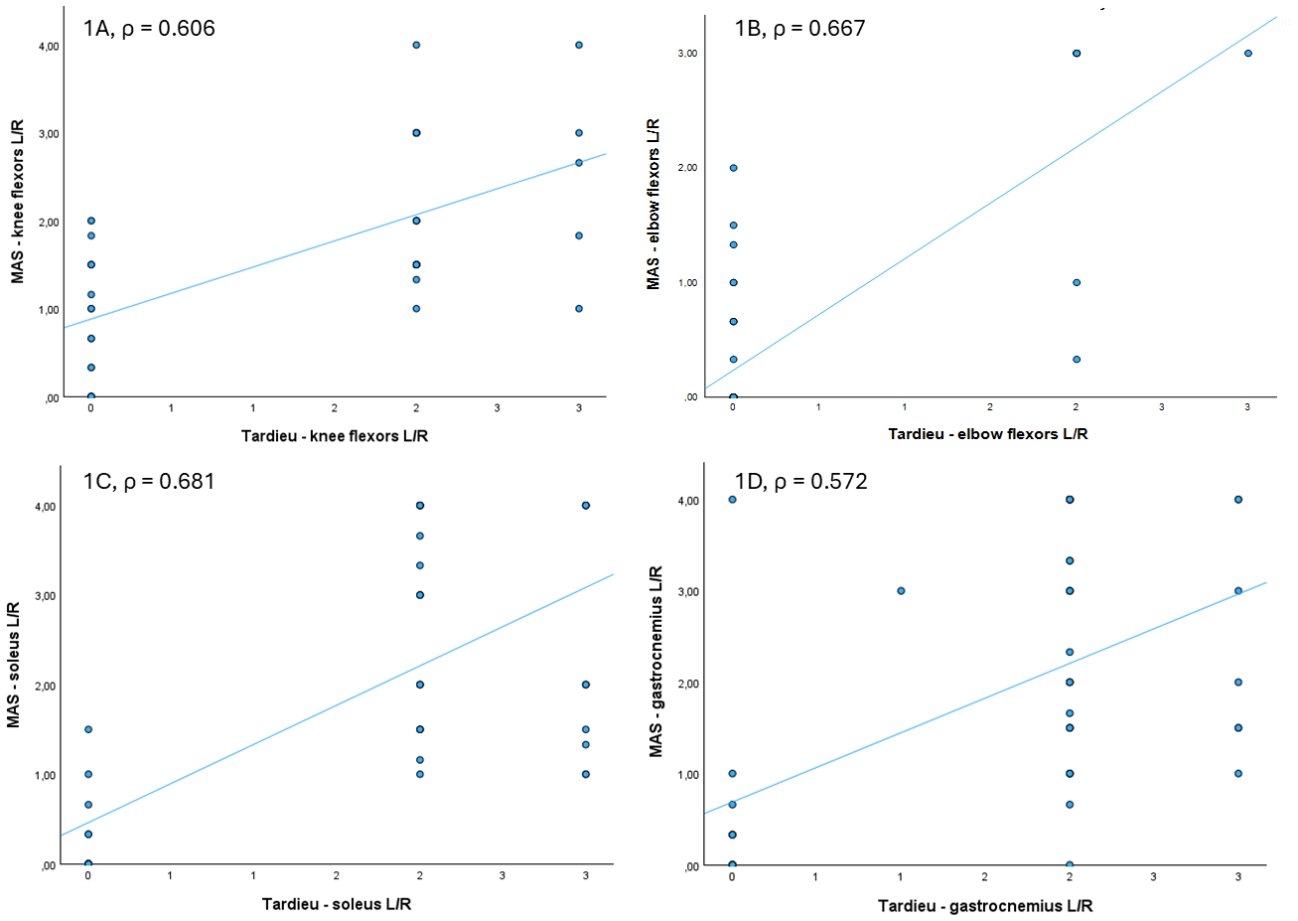
Scatter plots illustrating the relationship between Modified Ashworth Scale (MAS) scores and Tardieu Scale R2–R1 values for each examined muscle group. Spearman correlations for (A) knee flexors, (B) elbow flexors, (C) soleus, and (D) gastrocnemius. Each point represents a single muscle observation (left and right sides combined).

Interrater reliability was generally good for all muscles assessed. The lowest interrater agreement was found for knee flexors, with confidence intervals indicating reliability ranging from moderate to good, whereas plantar flexors demonstrated comparatively narrower confidence intervals, reflecting more consistent agreement. Table 2 presents the results of intra- and interrater reliability. Pairwise interrater comparisons (Table 3) showed variability across examiner combinations, with excellent agreement observed only in the assessment of the soleus muscle between examiners A and C (ICC = 0.91). Spearman correlation analysis demonstrated moderate positive associations between MAS scores and TS R2–R1 values across all examined muscle groups (Fig. 1).

Bland–Altman analysis was conducted to further examine interrater agreement patterns. For most muscle groups, the 95% confidence intervals of the mean differences included zero, indicating no statistically significant systematic bias between examiners. However, significant bias was observed in selected comparisons, particularly for the soleus muscle (examiners AB and AC), gastrocnemius muscle (AB), and elbow flexors (BC). Across muscle groups, limits of agreement were relatively wide, in several cases approaching ±1.5 to ±2 MAS grades. These findings indicate that while ICC estimates reflect good overall agreement at the group level, individual-level interchangeability between examiners may be limited in certain muscle groups.

## Discussion

The present study demonstrated excellent intrarater reliability (ICC > 0.90) of the MAS across all examined muscle groups. Interrater reliability was generally good (ICC = 0.80–0.89), although confidence intervals indicated moderate-to-good agreement in certain muscles, particularly knee flexors. These findings suggest that the MAS may provide reproducible measurements at the group level in a primary healthcare setting.

In this study, intrarater reliability for elbow flexor assessment was excellent (ICC > 0.90), whereas previous studies have reported reliability ranging from moderate to good (Clopton et al., 2005; Klingels et al., 2010). Variability across studies may reflect differences in examiner training, sample heterogeneity, and protocol standardization. Similarly, for knee flexors, previously reported intrarater reliability ranges from low to good (Numanoğlu & Günel, 2012; Clopton et al., 2005; Fosang et al., 2003), whereas our findings demonstrated consistently excellent agreement (lowest ICC = 0.91). Differences in limb lever length, muscle mass, stabilization technique, and behavioral characteristics of pediatric participants may contribute to variability in repeated measurements.

**Table 2:** ICC with 95% CI in intra- and interrater reliability.

	**Absolute agreement**				**Consistent agreement**	
	**Intrarater reliability**		**Interrater reliability**		**Interrater reliability**	
	ICC(3,k)	CI	ICC(2,k)	CI	ICC(2,k)	CI
Elbow flexors	0.99	0.97–0.99	0.87	0.78–0.93	0.88	0.81–0.93
Knee flexors	0.91	0.84–0.95	0.81	0.70–0.89	0.82	0.70–0.89
Soleus muscle	0.97	0.95–0.98	0.89	0.82–0.94	0.90	0.83–0.94
Gastrocnemius muscle	0.97	0.95–0.98	0.85	0.76–0.91	0.86	0.77–0.92

**Notes.**

ICC(3,k) two-way mixed-effects model, absolute agreement ICC(2,k): two-way random-effects model, absolute agreement. CI confidence interval ICC intraclass correlation coefficient

**Table 3:** ICC with 95% CI in interrater reliability (AB, AC and BC) with absolute agreement.

	**Elbow flexors**		**Knee flexors**		**Soleus muscle**		**Gastrocnemius muscle**	
	ICC	CI	ICC	CI	ICC	CI	ICC	CI
A B	0.76	0.57–0.87	0.72	0.50–0.84	0.77	0.58–0.87	0.72	0.48–0.85
A C	0.85	0.73–0.92	0.69	0.45–0.83	0.91	0.83–0.95	0.86	0.76–0.92
B C	0.84	0.47–0.94	0.85	0.73–0.92	0.84	0.71–0.91	0.77	0.59–0.87

**Notes.**

ICC intraclass correlation coefficient CI confidence interval

For plantar flexors, intrarater reliability in earlier studies varies substantially. Soleus muscle reliability has been reported as low to good (Numanoğlu & Günel, 2012; Salem et al., 2010), and gastrocnemius reliability from low to moderate (Mutlu, Livanelioglu & Gunel, 2008; Fosang et al., 2003). Methodological differences, including retest intervals and number of repetitions, may partly explain this variability. For example, Fosang et al. (2003) used a 3–5 day retest interval, whereas Salem et al. (2010) did not specify the interval. Shorter retest intervals may increase the potential influence of recall effects (Nomikos, Spence & Alshehri, 2018). Furthermore, repetition protocols may influence the stability of MAS scoring, as repeated passive stretches can transiently modulate stretch reflex responses (Clopton et al., 2005).

Interrater reliability in this study was generally good (ICC = 0.80−0.89), consistent with findings reported by Clopton et al. (2005) and Mutlu, Livanelioglu & Gunel (2008). Some studies have reported lower interrater agreement (Fosang et al., 2003; Klingels et al., 2010), which may be attributable to differences in protocol standardization, examiner experience, or population characteristics. In our sample, a substantial proportion of participants presented with milder functional impairment according to GMFCS classification, which may have facilitated clearer differentiation of resistance during passive movement. In populations with more severe impairment, greater variability in muscle tone expression may complicate scoring.

The greatest variability in the literature is observed in soleus assessment. Clopton et al. (2005) reported low interrater reliability, potentially reflecting the inclusion of a heterogeneous clinical population. Variability in examiner training has also been highlighted as a determinant of scoring consistency (Yam & Leung, 2006). In this study, predefined stabilization techniques and examiner standardization procedures may have contributed to higher reliability estimates. Nevertheless, complete uniformity in force application, stretch direction, and velocity is difficult to achieve in clinical practice, which may partly explain differences across studies.

Mutlu, Livanelioglu & Gunel (2008) based MAS scoring on a single repetition, whereas in this study the outcome represented the average of three repetitions. Averaging may enhance score stability; however, this interpretation remains speculative and would require direct methodological comparison.

Importantly, Bland–Altman analysis in the present study demonstrated that, despite good ICC values, limits of agreement were relatively wide in several muscle groups (approximately ±1–2 MAS grades), and small but statistically significant examiner-dependent differences were observed in selected comparisons. These findings indicate that MAS demonstrates acceptable reliability at the group level, but individual-level interchangeability between examiners may be limited. This observation is consistent with previous concerns regarding the ordinal structure and subjective grading characteristics of the MAS (Fosang et al., 2003).

A moderate association between TS and MAS was observed in our study (*ρ* = 0.578−0.69). However, the two scales measure related but distinct constructs. MAS reflects resistance to passive movement without distinguishing neural from mechanical contributions, whereas TS specifically captures the velocity-dependent component of muscle response (Gracies et al., 2010). Therefore, the observed association between MAS and TS should not be interpreted as evidence of criterion or concurrent validity, but rather as reflecting partial overlap between resistance-based and velocity-dependent manifestations of hypertonia. Additionally, assigning a value of 0 degrees in cases where no catch was detected reflects the absence of a measurable dynamic component; however, this approach may reduce variability at the lower end of TS scores and potentially attenuate the observed association.

Several limitations should be considered when interpreting these findings. The heterogeneous clinical characteristics of the sample, including variability in functional severity and distribution of spasticity, may have influenced reliability estimates and limited comparability with studies using more homogeneous populations (Shevell et al., 2008). Although all examined muscles were analyzed collectively to reflect real-world primary healthcare practice, subgroup-specific reliability may differ.

Although a 7–14-day interval was chosen to minimize immediate recall effects, some degree of assessor recall cannot be entirely excluded and may have contributed to higher intrarater agreement (Nomikos, Spence & Alshehri, 2018). The participant used during examiner familiarization was assessed approximately one month before formal data collection; while this interval was considered sufficient to reduce recall bias, it cannot be completely excluded.

An additional limitation relates to the cumulative number of passive stretches performed during interrater assessment. Although the MAS assessments were separated by five-minute intervals, repeated fast stretches within a single session may transiently influence spasticity expression through short-term reflex modulation or viscoelastic adaptation. This methodological constraint represents a broader challenge inherent to interrater spasticity assessment in clinical research (Clopton et al., 2005).

Another limitation concerns the timing of the TS assessment. TS was performed after the MAS testing to prevent high velocity stretches from influencing MAS scoring. However, repeated passive stretching during the MAS assessment may have transiently altered muscle tone, and the TS measurements obtained thereafter may not fully reflect baseline spasticity. Although the interval exceeded the duration of immediate stretch-induced reflex inhibition described in neurophysiological studies, cumulative effects cannot be entirely excluded (Gracies et al., 2010).

Finally, the ordinal nature of the MAS represents a limitation when applying ICC, which assumes interval-level measurement. However, ICC was retained to ensure comparability with prior MAS reliability studies (Koo & Li, 2016). Although ordinal agreement statistics such as weighted kappa may provide complementary information, they were not included due to the small sample size and the presence of multiple raters and muscle groups, which would likely result in unstable and difficult-to-interpret estimates. Therefore, reliability results should be interpreted with appropriate caution.

Despite these limitations, the present findings suggest that MAS may be a practical and feasible tool for assessing spasticity in children with cerebral palsy in primary healthcare settings. With appropriate standardization and examiner training (Yam & Leung, 2006), MAS can provide reproducible group-level measurements. However, individual-level interpretation should remain cautious, particularly when monitoring subtle changes over time or comparing scores between different examiners. Future research should include larger and more homogeneous samples, further protocol standardization regarding stretch speed and repetition number, and exploration of adjunct objective measurement strategies. For example, metronome-guided movement speed has been proposed to improve standardization of ankle plantar flexor assessment (Yoo & Lim, 2022).

## Conclusions

This study found that the MAS demonstrated excellent intrarater and generally good interrater reliability for assessing spasticity in children with CP in a primary healthcare setting. However, Bland–Altman analysis indicates that individual-level agreement between examiners may vary in certain muscle groups. A moderate association between the MAS and the TS was observed, reflecting partial overlap between resistance-based and velocity-dependent components of hypertonia rather than criterion validity. The MAS appears to be a feasible clinical tool for group-level assessment in a heterogeneous primary-level population when standardized procedures are applied. However, cautious interpretation is warranted at the individual level, particularly when monitoring subtle changes or comparing scores between examiners. Further research involving larger and more homogeneous samples, as well as additional protocol standardization, is recommended to enhance comparability and clinical applicability.

##  Supplemental Information

10.7717/peerj.21349/supp-1 Supplemental Information 1 Operational definitions of Modified Ashworth Scale grades * Note:* Grades were applied according to the original operational definitions described by Bohannon & Smith (1987) and clarified by Pandyan et al. (1999), without modification.

10.7717/peerj.21349/supp-2 Supplemental Information 2 Concurent validity of MAS *vs* TS dataset

10.7717/peerj.21349/supp-3 Supplemental Information 3 Inerrater variability MAS (3 asessors) dataset

10.7717/peerj.21349/supp-4 Supplemental Information 4 Intra-rater reliability (MAS) raw data

10.7717/peerj.21349/supp-5 Supplemental Information 5 STROBE checklist

## Additional Information and Declarations

### Data Availability

The following information was supplied regarding data availability: The raw data are available in the Supplemental Files.
