# Supplementary material for: Intra- and interrater reliability of the Modified Ashworth Scale and its association with the Tardieu Scale in children with cerebral palsy
Source: PeerJ. 2026 Jul 1;14:e21349. doi: 10.7717/peerj.21349 (PMC13332714; doi:10.7717/peerj.21349)
Supplement: Supplemental Information 1 — Note: Grades were applied according to the original operational definitions described by Bohannon & Smith (1987) and clarified by Pandyan et al. (1999), without modification. [file peerj-14-21349-s001.docx]

# Supplementary table S1: Operational definitions of the MAS grades

| **Grade** | **Operational definition (as applied in this study)** |
| --- | --- |
| **0** | No increase in muscle tone. |
| **1** | Slight increase in muscle tone, manifested by a catch and release or by minimal resistance at the end of the range of motion. |
| **1+** | Slight increase in muscle tone, manifested by a catch followed by minimal resistance through less than half of the range of motion. |
| **2** | More marked increase in muscle tone through most of the range of motion, but the affected part is easily moved. |
| **3** | Considerable increase in muscle tone; passive movement is difficult. |
| **4** | Affected part rigid in flexion or extension. |

*Note:* Grades were applied according to the original operational definitions described by Bohannon and Smith (1987) and clarified by Pandyan et al. (1999), without modification.
