# Supplementary material for: Intra- and interrater reliability of the Modified Ashworth Scale and its association with the Tardieu Scale in children with cerebral palsy
Source: PeerJ. 2026 Jul 1;14:e21349. doi: 10.7717/peerj.21349 (PMC13332714; doi:10.7717/peerj.21349)
Supplement: Supplemental Information 5 [file peerj-14-21349-s005.doc]

STROBE Statement—Checklist of items that should be included in reports of ***cross-sectional studies***

|  | Item No | Recommendation / Responses in blue |
| --- | --- | --- |
| **Title and abstract** | 1 | (*a*) Indicate the study’s design with a commonly used term in the title or the abstract  Provided in the abstract (cross-sectional study, page 2, line 21) |
|  |  | (*b*) Provide in the abstract an informative and balanced summary of what was done and what was found Structured abstract provided (page 2, lines 16 – 33) |
| Introduction | | |
| Background/rationale | 2 | Explain the scientific background and rationale for the investigation being reported  Provided in detail (p 2- 4, lines 36 – 113) |
| Objectives | 3 | State specific objectives, including any prespecified hypotheses  Provided p 4, lines 114 – 122) |
| Methods | | |
| Study design | 4 | Present key elements of study design early in the paper  Provided (p 4, line 125) |
| Setting | 5 | Describe the setting, locations, and relevant dates, including periods of recruitment, exposure, follow-up, and data collection  Provided (p 5, lines 152-161) |
| Participants | 6 | (*a*) Give the eligibility criteria, and the sources and methods of selection of participants Provided (p 4-5, lines 128 – 142) |
| Variables | 7 | Clearly define all outcomes, exposures, predictors, potential confounders, and effect modifiers. Give diagnostic criteria, if applicable Provided (p 5, lines 152-161) |
| Data sources/ measurement | 8* | For each variable of interest, give sources of data and details of methods of assessment (measurement). Describe comparability of assessment methods if there is more than one group Provided (p 5-6, lines 164-202) |
| Bias | 9 | Describe any efforts to address potential sources of bias  Described in the measurement procedure |
| Study size | 10 | Explain how the study size was arrived at  Sample size justified based on feasibility and comparable studies. Power calculation rationale provided (detecting moderate effect sizes). |
| Quantitative variables | 11 | Explain how quantitative variables were handled in the analyses. If applicable, describe which groupings were chosen and why |
| Statistical methods | 12 | Describe all statistical methods, including those used to control for confounding |
|  |  | (*b*) Describe any methods used to examine subgroups and interactions |
|  |  | (*c*) Explain how missing data were addressed |
|  |  | (*d*) If applicable, describe analytical methods taking account of sampling strategy |
|  |  | (*e*) Describe any sensitivity analyses  All applicable information are provided in Data analysis subsection of the Methods (p 6, lines 205-223) |
| Results | | |
| Participants | 13* | (a) Report numbers of individuals at each stage of study—eg numbers potentially eligible, examined for eligibility, confirmed eligible, included in the study, completing follow-up, and analysed  Final sample includes 24 participants, and all participants completed the assessment. |
|  |  | (b) Give reasons for non-participation at each stage NA |
|  |  | (c) Consider use of a flow diagram / |
| Descriptive data | 14* | (a) Give characteristics of study participants (eg demographic, clinical, social) and information on exposures and potential confounders |
|  |  | (b) Indicate number of participants with missing data for each variable of interest NA |
| Outcome data | 15* | Report numbers of outcome events or summary measures  Outcome data are presented clearly in Tables in result section. |
| Main results | 16 | (*a*) Give unadjusted estimates and, if applicable, confounder-adjusted estimates and their precision (eg, 95% confidence interval). Make clear which confounders were adjusted for and why they were included NA |
|  |  | (*b*) Report category boundaries when continuous variables were categorized / |
|  |  | (*c*) If relevant, consider translating estimates of relative risk into absolute risk for a meaningful time period NA |
| Other analyses | 17 | Report other analyses done—eg analyses of subgroups and interactions, and sensitivity analyses NA |
| Discussion | | |
| Key results | 18 | Summarise key results with reference to study objectives  Summarized in the discussion section (p 7-9). |
| Limitations | 19 | Discuss limitations of the study, taking into account sources of potential bias or imprecision. Discuss both direction and magnitude of any potential bias  Provided (p 9, lines 318 – 328) |
| Interpretation | 20 | Give a cautious overall interpretation of results considering objectives, limitations, multiplicity of analyses, results from similar studies, and other relevant evidence  Provided (p 9, lines 313 – 318 and 328 - 330) |
| Generalisability | 21 | Discuss the generalisability (external validity) of the study results  Provided (p 10, lines 353 - 355) |
| Other information | | |
| Funding | 22 | Give the source of funding and the role of the funders for the present study and, if applicable, for the original study on which the present article is based  We did not receive any funding for conduction of this study. |

*Give information separately for exposed and unexposed groups.

**Note:** An Explanation and Elaboration article discusses each checklist item and gives methodological background and published examples of transparent reporting. The STROBE checklist is best used in conjunction with this article (freely available on the Web sites of PLoS Medicine at http://www.plosmedicine.org/, Annals of Internal Medicine at http://www.annals.org/, and Epidemiology at http://www.epidem.com/). Information on the STROBE Initiative is available at www.strobe-statement.org.
